# Multisite Study of the Management of Musculoskeletal Infection After Trauma: The MMUSKIT Study

**DOI:** 10.1093/ofid/ofae262

**Published:** 2024-05-06

**Authors:** Jessica Seidelman, Alaina S Ritter, Emily Poehlein, Cynthia L Green, Damon V Briggs, Tristan Chari, Aaron D Therien, Alexandra Hunter Aitchison, Kiera Lunn, Christian F Zirbes, Tanvi Manohar, Diana V Rijo, Jennifer E Hagen, Michael T Talerico, Malcolm R DeBaun, Christian A Pean, Laura Certain, Sandra B Nelson

**Affiliations:** Division of Infectious Diseases and International Health, Department of Medicine, School of Medicine, Duke University, Durham, North Carolina, USA; Division of Infectious Diseases and Global Medicine, College of Medicine, University of Florida, Gainesville, Florida, USA; Department of Biostatistics and Bioinformatics, School of Medicine, Duke University, Durham, North Carolina, USA; Department of Biostatistics and Bioinformatics, School of Medicine, Duke University, Durham, North Carolina, USA; School of Medicine, Duke University, Durham, North Carolina, USA; School of Medicine, Duke University, Durham, North Carolina, USA; School of Medicine, Duke University, Durham, North Carolina, USA; School of Medicine, Duke University, Durham, North Carolina, USA; School of Medicine, Duke University, Durham, North Carolina, USA; School of Medicine, Duke University, Durham, North Carolina, USA; Division of Infectious Diseases and Global Medicine, College of Medicine, University of Florida, Gainesville, Florida, USA; Department of Orthopedics and Sports Medicine, University of Florida, Gainesville, Florida, USA; Department of Orthopedics and Sports Medicine, University of Florida, Gainesville, Florida, USA; Department of Orthopedics and Sports Medicine, University of Florida, Gainesville, Florida, USA; Department of Orthopaedic Surgery, School of Medicine, Duke University, Durham, North Carolina, USA; Department of Orthopaedic Surgery, School of Medicine, Duke University, Durham, North Carolina, USA; Division of Infectious Diseases, University of Utah, Salt Lake City, Utah, USA; Division of Infectious Diseases, Department of Medicine, Massachusetts General Hospital, Boston, Massachusetts, USA; Harvard Medical School, Boston, Massachusetts, USA

**Keywords:** fracture-related infection, infection after fracture fixation

## Abstract

**Background:**

The optimal duration and choice of antibiotic for fracture-related infection (FRI) is not well defined. This study aimed to determine whether antibiotic duration (≤6 vs >6 weeks) is associated with infection- and surgery-free survival. The secondary aim was to ascertain risk factors associated with surgery- and infection-free survival.

**Methods:**

We performed a multicenter retrospective study of patients diagnosed with FRI between 2013 and 2022. The association between antibiotic duration and surgery- and infection-free survival was assessed by Cox proportional hazard models. Models were weighted by the inverse of the propensity score, calculated with a priori variables of hardware removal; infection due to *Staphylococcus aureus*, *Staphylococcus lugdunensis*, *Pseudomonas* or *Candida* species; and flap coverage. Multivariable Cox proportional hazard models were run with additional covariates including initial pathogen, need for flap, and hardware removal.

**Results:**

Of 96 patients, 54 (56.3%) received ≤6 weeks of antibiotics and 42 (43.7%) received >6 weeks. There was no association between longer antibiotic duration and surgery-free survival (hazard ratio [HR], 0.95; 95% CI, .65–1.38; *P* = .78) or infection-free survival (HR, 0.77; 95% CI, .30–1.96; *P* = .58). Negative culture was associated with increased hazard of reoperation or death (HR, 3.52; 95% CI, 1.99–6.20; *P* < .001) and reinfection or death (HR, 3.71; 95% CI, 1.24–11.09; *P* < .001). Need for flap coverage had an increased hazard of reoperation or death (HR, 3.24; 95% CI, 1.61–6.54; *P* = .001).

**Conclusions:**

The ideal duration of antibiotics to treat FRI is unclear. In this multicenter study, there was no association between antibiotic treatment duration and surgery- or infection-free survival.

## Teaser

Longer courses of antibiotics (>6 vs ≤6 weeks) were not significantly associated with surgery- or infection-free survival among patients with infection after fracture fixation.

## BACKGROUND

Fracture-related infection (FRI) is a challenging complication of musculoskeletal trauma and can lead to serious morbidity and mortality [1, 2]. The incidence of FRI varies depending on injury severity, with rates ranging from 1.8% for closed fractures to 27% for open fractures [3]. FRI cases generate 6.5-times higher health care costs as compared with noninfected cases [3]. The increased cost and morbidity attributed to FRI are a consequence of the need for multiple surgical procedures, antibiotic treatment, and prolonged rehabilitation [4]. Treatment of FRI typically requires multiple surgical procedures and prolonged antibiotic therapy, and it still has a high rate of recurrence [1]. Success of treatment for FRI is not optimal; in one study, treatment success was only 70% with a recurrence rate of 9% and an amputation rate of 3% [3].

Unfortunately, the optimal treatment of FRI is unknown, particularly regarding choice and duration of antibiotic therapy. Some authors recommend 6 to 12 weeks of antibiotic therapy depending on the type and severity of infection, the presence of implants, and the response to treatment [5, 6]. There is a lack of evidence, however, to support this recommendation. In fact, some data suggest that shorter courses of antibiotic therapy for bone and joint infection may be equally effective and less harmful than longer courses [7].

This study pooled observational data from multiple academic medical centers to assess whether differences in antibiotic treatment duration are associated with outcomes for the treatment of FRI. The primary aim of our study was to determine whether antibiotic duration ≤6 vs >6 weeks is associated with infection- and surgery-free survival. A secondary aim was to assess factors associated with infection- and surgery-free survival. We also assessed whether concern for infection at the time of reoperation is associated with antibiotic duration among patients undergoing repeat operation. Last, we reviewed patients with recurrent infection at the time of reoperation to determine whether antibiotic duration correlates with identification of a new pathogen at reoperation.

## METHODS

We performed a retrospective cohort study among 4 academic medical centers. All 4 hospitals are level 1 trauma centers and employ fellowship-trained orthopedic trauma surgeons and infectious disease physicians with expertise in orthopedic infections.

Each site identified potential patients via *CPT* codes for debridement that occurred between July 2013 and February 2022. Patients were included if they were aged ≥18 years, underwent open reduction and internal fixation (ORIF) for long bone trauma, subsequently had debridement surgery due to infection between 14 days and 6 months after fixation, and received at least 2 weeks of postoperative antibiotics after debridement surgery. Patients who developed reinfection or underwent reoperation within 6 weeks of the initial debridement surgery were excluded. Patient and surgery variables were collected by a standardized data collection tool in REDCap [8] (Supplementary Figure 1).

The primary outcomes were surgery- and infection-free survival. Secondary outcomes included return to the operating room for suspected infection and identification of the same or different pathogen during the subsequent operation. The primary exposure of interest was antibiotic duration, which was dichotomized at 6 weeks [7, 9]. Patients receiving a few days beyond 6 weeks (eg, to accommodate clinic schedules) were included in the ≤6-week group.

Descriptive baseline and surgical characteristics were presented overall and by duration of antibiotics. Continuous variables were displayed as mean (SD) or median (IQR) if skewed. Categorical variables were presented as counts with percentage of nonmissing values.

Cox proportional hazard models for surgery- and infection-free survival were run with antibiotic duration as the primary predictor. Hazard ratios (HRs) with associated 95% CIs and *P* values are reported with weighted Kaplan-Meier survival plots. To address imbalance of prognostic factors between the treatment groups, models were weighted by the inverse of the propensity score, which was estimated by logistic regression with a priori prognostic variables of hardware removal (all vs partial/none); infection with *Staphylococcus aureus*, *Staphylococcus lugdunensis*, *Pseudomonas* spp, or *Candida* spp; and need for flap. Success of the propensity score to balance covariates was assessed by standardized mean differences with a value <0.10 considered negligible. A random intercept was included to account for correlated outcomes and treatment practices by institution. The proportional hazard assumption was assessed by testing the interaction of each covariate with time.

To assess factors associated with infection- and surgery-free survival, additional covariates that may affect selected antimicrobial duration (initial pathogen, flap coverage, and hardware removal) were included in the Cox proportional hazard models.

To assess whether reoperation for suspected infection is associated with antibiotic duration among patients with a repeat procedure, logistic regression was used with concern for infection as the outcome and antibiotic duration as the primary predictor. The indication for return to the operating room was based on documentation in the medical record and included suspected infection, hardware failure, repeat fracture/trauma, painful hardware, nonunion, need for soft tissue infection, or other reason (Supplementary Figure 1). The odds ratio for antibiotic duration is reported with associated 95% CI and *P* value. To assess whether antibiotic duration was associated with infection with a new pathogen or the same pathogen, 2 logistic regression models were used with infection type as the outcome (new vs not new; same vs not same) and antibiotic duration as the primary predictor.

All analyses were conducted with SAS version 9.4 (SAS Institute), and *P* < .05 was considered statistically significant.

The institutional review board at each academic institution approved this research study.

## RESULTS

**Figure 1.**
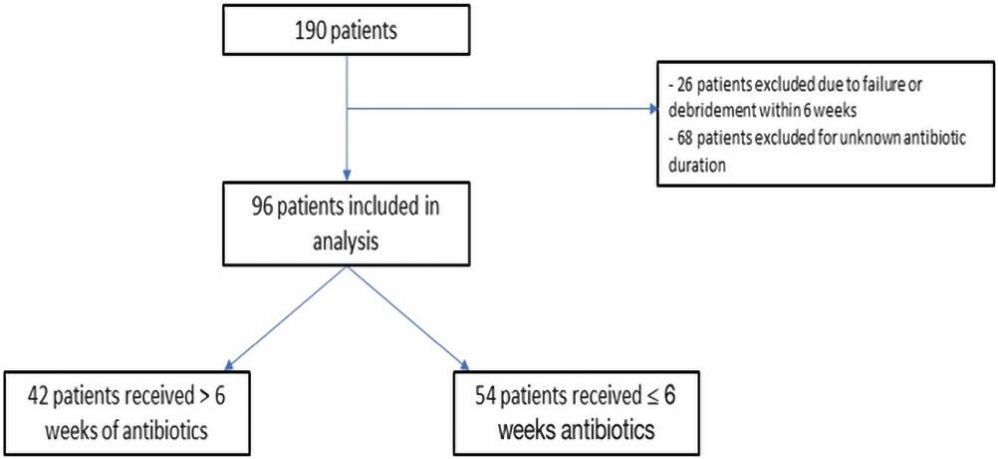
Inclusion/exclusion criteria.

An overall 190 patients met inclusion criteria and were eligible for the study (Figure 1). Of these patients, 26 were excluded due to failure within 6 weeks or having <6 weeks of follow-up from debridement, and an additional 68 patients were excluded for unknown antibiotic duration (eg, including those on indefinite antibiotic suppression). Therefore, 96 patients were included in the primary analysis. Of these, 54 (56.3%) received ≤6 weeks of antibiotic therapy, while the remaining 42 (43.7%) received an antibiotic duration >6 weeks.

The demographic and clinical characteristics of the study cohort at the time of debridement surgery, stratified by antibiotic duration, are provided in Table 1. Demographic and clinical characteristics of the study groups at the time of the original ORIF, stratified by antibiotic duration, hospital, and outcome, are provided in Supplementary Tables 1 and 2. A higher percentage of patients receiving ≤6 weeks of antibiotics were male as compared with patients receiving >6 weeks of antibiotics (61.1% vs 54.8%). Most patients had a grade II (n = 41, 43.6%) or III (n = 42, 44.7%) American Society of Anesthesiologists score. The most commonly fractured bone was the tibia (n = 81, 84.4%). The median (IQR) number of days from ORIF to debridement was longer for patients receiving ≤6 weeks of antibiotics vs >6 weeks (67 [27–103] vs 40 [27–78] days). The median number of days from debridement to last documentation in the electronic medical record was greater among patients with >6 weeks of antibiotics vs ≤6 weeks (629 [228–1239] vs 4652 [231–1119] days).

**Table 1.** Characteristics of Patients at the Time of Debridement Surgery for Infection

	Time of Surgery		
	≤6 wk (n = 54)	>6 wk (n = 42)	Total (N = 96)
Days from ORIF to debridement surgery	67.0 (27.0–103.0)	40.0 (27.0–78.0)	48.0 (27.0–95.5)
Age, y	51.6 ± 17.2	47.4 ± 18.2	49.8 ± 17.7
Body mass index, kg/m^2^	29.8 ± 8.1	29.2 ± 6.8	29.5 ± 7.5
Male	33 (61.1)	23 (54.8)	56 (58.3)
Diabetes mellitus	15 (27.8)	8 (19.0)	23 (24.0)
American Society of Anesthesiologists grade			
Missing	2 (3.7)	0 (.)	2 (3.7)
I	4 (7.7)	5 (11.9)	9 (9.6)
II	24 (46.2)	17 (40.5)	41 (43.6)
III	22 (42.3)	20 (47.6)	42 (44.7)
IV	2 (3.8)	0 (0.0)	2 (2.1)
Smoking status			
Current smoker	19 (35.2)	9 (21.4)	28 (29.2)
Prior smoker	14 (25.9)	14 (33.3)	28 (29.2)
Never smoker	20 (37.0)	19 (45.2)	39 (40.6)
Unknown	1 (1.9)	0 (0.0)	1 (1.0)
Immunosuppressed	3 (5.6)	0 (0.0)	3 (3.1)
Laboratory results^a^			
C-reactive protein, mg/dL	5.5 (1.6–19.0)	7.8 (1.4–23.6)	6.8 (1.5–19.2)
Erythrocyte sedimentation rate, mm/h	42.0 (20.0–79.0)	46.0 (30.0–75.0)	43.5 (23.0–77.0)
White blood cells, 10^9^/L	8.6 (7.2–11.2)	8.0 (6.2–9.5)	8.6 (6.7–10.5)
Albumin, g/dL	3.4 (3.2–3.9)	3.6 (3.2–3.9)	3.5 (3.2–3.9)
No. of surgical procedures required to manage first infection episode	1.0 (1.0–3.0)	2.0 (1.0–3.0)	2.0 (1.0–3.0)
Pathogens isolated from infection surgery			
Aerobic gram-positive organisms			
Methicillin-resistant *Staphylococcus aureus*	12 (22.2)	5 (11.9)	17 (17.7)
Methicillin-susceptible *Staphylococcus aureus*	14 (25.9)	9 (21.4)	23 (24.0)
*Staphylococcus lugdunensis*	1 (1.9)	0 (0.0)	1 (1.0)
Other coagulase-negative staphylococci	11 (20.4)	5 (11.9)	16 (16.7)
Enterococcus species	7 (13.0)	6 (14.3)	13 (13.5)
Other aerobic gram-positive cocci	5 (9.3)	4 (9.5)	9 (9.4)
Aerobic gram-negative organisms			
Pseudomonas species	5 (9.3)	4 (9.5)	9 (9.4)
Other gram-negative bacilli	22 (40.7)	21 (50.0)	43 (44.8)
Anaerobes			
*Cutibacterium* species	0 (0.0)	1 (2.4)	1 (1.0)
Other anaerobes	9 (16.7)	9 (21.4)	18 (18.8)
*Candida* species/mold	2 (3.7)	1 (2.4)	3 (31)
Nontuberculous mycobacteria	0 (0.0)	1 (2.4)	1 (1.0)
Culture negative	4 (7.4)	4 (9.5)	8 (8.3)
No culture collected	0 (0.0)	1 (2.4)	1 (1.0)
MDROs^b^	7 (13.0)	3 (7.1)	10
Topical antibiotics used^c^	24 (44.4)	24 (57.1)	48 (50.0)
Vancomycin	21 (38.9)	22 (52.4)	43 (44.8)
Tobramycin/gentamicin	14 (25.9)	12 (28.6)	26 (27.1)
Ceftazidime	0 (0.0)	7 (16.7)	7 (7.3)
Other	3 (5.6)	3 (7.1)	6 (6.3)
Unknown	1 (1.9)	2 (4.8)	3 (3.1)
Hardware removed			
All	22 (40.7)	9 (21.4)	31 (32.3)
None	23 (42.6)	23 (54.8)	46 (47.9)
Partial removal	9 (16.7)	10 (23.8)	19 (19.8)
New hardware placed or replaced			
Yes	14 (26.4)	6 (14.3)	20 (21.1)
No	39 (73.6)	36 (85.7)	75 (78.9)
Antibiotic received during initial antibiotic course			
Amoxicillin/amoxicillin-clavulanate	3 (5.6)	1 (2.4)	4 (4.2)
Ampicillin-sulbactam/piperacillin-tazobactam	3 (5.6)	4 (9.5)	7 (7.3)
Cefazolin	7 (13.0)	5 (11.9)	12 (12.5)
Third/fourth-generation cephalosporin	13 (24.1)	14 (33.3)	27 (28.1)
Ciprofloxacin/levofloxacin	11 (20.4)	21 (50.0)	32 (33.3)
Clindamycin	0 (0.0)	1 (2.4)	1 (1.0)
Daptomycin/vancomycin	29 (53.7)	13 (31.0)	42 (43.8)
Doxycycline	2 (3.7)	2 (4.8)	4 (4.2)
Carbapenem	9 (16.7)	2 (4.8)	11 (11.5)
Linezolid	1 (1.9)	1 (2.4)	2 (2.1)
Metronidazole	4 (7.4)	3 (7.1)	7 (7.3)
Nafcillin/oxacillin	2 (3.7)	0 (0.0)	2 (2.1)
Trimethoprim-sulfamethoxazole	3 (5.6)	4 (9.5)	7 (7.3)
Other	3 (5.6)	2 (4.8)	5 (5.2)
Total weeks of antibiotics	6.38 ± 1.04	16.22 ± 8.80	10.68 ± 7.62

Data are presented as No. (%), mean ± SD, or median (IQR).

Abbreviations: MDRO, multidrug-resistant organism; ORIF, open reduction and internal fixation.

^a^Laboratory values were recorded 2 weeks before or after initial infection surgery. If multiple values existed, the value closest to the date of surgery was recorded.

^b^Organisms in this category were vancomycin-resistant enterococcus, carbapenem-resistant Enterobacteriaceae, extended-spectrum β-lactamase Enterobacteriaceae, and multidrug-resistant organisms as defined by nonsusceptibility (ie, resistant or intermediate) to at least 1 agent in at least 3 antimicrobial classes of the following 6 classes: ampicillin/sulbactam, cephalosporins (cefepime, ceftazidime), β-lactam/β-lactam β-lactamase inhibitor combination (piperacillin, piperacillin/tazobactam), carbapenems (imipenem, meropenem, doripenem), fluoroquinolones (ciprofloxacin or levofloxacin), and aminoglycosides (gentamicin, tobramycin, or amikacin).

^c^Topical antibiotics that were used during the first infection operation and not the original ORIF.

**Table 2.** Cox Proportional Hazard Model Results for Risk Factors of Surgery- and Infection-Free Survival

	Surgery-Free Survival, HR (95% CI)	*P* Value	Infection-Free Survival, HR (95% CI)	*P* Value
Need for flap	3.24 (1.61–6.54)	.001	1.62 (.35–7.25)	.54
All hardware removed	0.85 (.59–1.22)	.37	0.81 (.29–2.29)	.69
Open/closed	2.24 (.96–5.25)	.06	0.71 (.28–1.79)	.46
Staphylococcal infection	0.70 (.30–1.63)	.41	0.70 (.39–1.25)	.22
Negative culture	3.52 (1.99–6.20)	<.001	3.71 (1.24–11.09)	.019

Abbreviations: HR, hazard ratio.

Prior to propensity adjustment, patients who received >6 weeks of antibiotics were less likely to have all hardware removed (21.4% vs 40.7%), less likely to have *S aureus* or *Pseudomonas* spp infection (42.9% vs 59.3%), and slightly more likely to need flap coverage (16.7% vs 13.0%). Furthermore, patients with *S aureus* or *Pseudomonas* spp infection were more likely to have hardware removed as compared with infections with different pathogens (40.0% vs 23.9%). The distributions of propensity scores between the treatment arms were comparable. After weighting, all standardized mean differences were negligible (Supplementary Figure 2).

**Figure 2.**
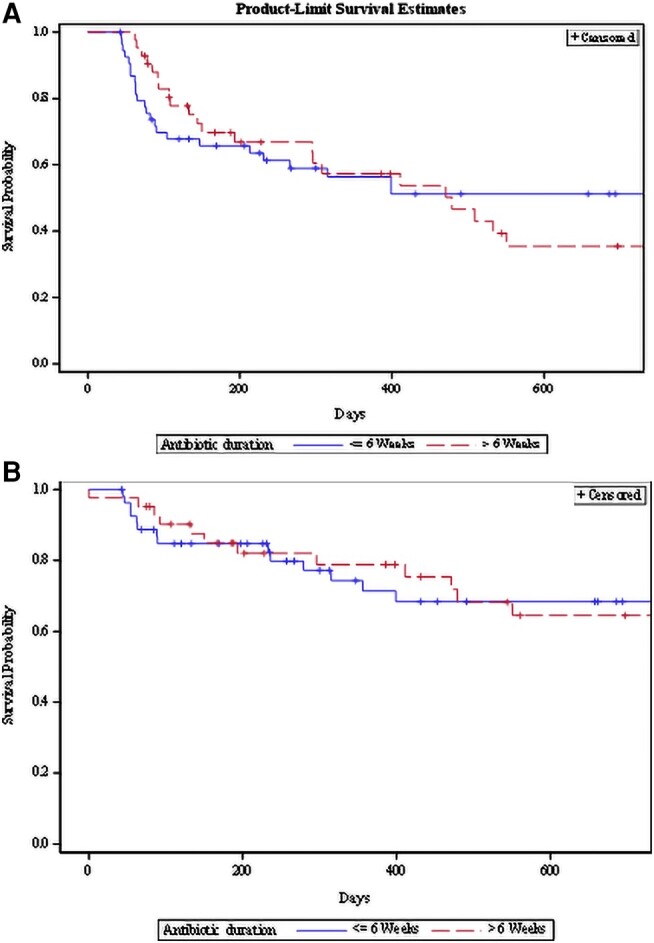
Kaplan-Meier curve of surgery- and infection-free survival between antibiotic duration groups.

Among the 96 patients, 47 (45.0%) returned to the operating room following initial treatment for infection, 25 (26.0%) had a reinfection, and 14 (14.6%) died during follow-up. After weighting by the inverse propensity score, there was no association between antibiotic duration and surgery-free survival (HR, 0.95; 95% CI, .65–1.38; *P* = .78) or infection-free survival (HR, 0.77; 95% CI, .30–1.96; *P* = .58; Figure 2).

We examined risk factors for infection- and surgery-free survival. Prior flap coverage was associated with increased hazard of return to the operating room or death (HR, 3.24; 95% CI, 1.61–6.54; *P* = .001). Negative culture was associated with an increased hazard of reoperation or death (HR, 3.52; 95% CI, 1.99–6.20; *P* < .001) and reinfection or death (HR, 3.71; 95% CI, 1.24–11.09; *P* = .019). Hardware removal, open fracture, and staphylococcal infection were not associated with surgery- or infection-free survival.

Concern for infection as the indication for reoperation was slightly more common among patients with ≤6 weeks of antibiotics vs those with >6 weeks (n = 15 [60.0%] vs n = 11 [50.0%], *P* = .39). Of the 26 patients who underwent reoperation because of concern for infection, 15 had a new pathogen identified, and 11 had the same pathogen identified (4 had new and same pathogens identified). The distribution of patients with a new pathogen was similar between the patients who received ≤6 or >6 weeks of antibiotics (odds ratio, 0.80; 95% CI, .17–3.86; *P* = .70).

## DISCUSSION

This is the first multicenter study in the United States investigating the optimal treatment duration of antibiotics following FRI. Examining only patients with a defined antibiotic course (ie, no indefinite suppression), our study did not find an association between antibiotic duration after surgical management of FRI and surgery- or infection-free survival. This reinforces the growing body of literature suggesting that shorter antibiotic durations may be as effective as longer durations for the treatment of bone and joint infections [7, 10–15]. Of note, none of these previous studies exclusively examined patients with FRI.

Studies examining the antibiotic treatment of FRI are challenging. Despite a 6-year study period and inclusion of 4 academic level 1 trauma centers, we identified only 96 patients who met inclusion criteria. We determined that *CPT* code utilization is a relatively poor identifier for FRI (Supplementary Figure 2). Common reasons why patients were excluded after identification of an appropriate *CPT* code included not having an infection that involved the fracture site, insufficient duration of follow-up evaluation after debridement surgery, lack of clear documentation of antibiotic duration, and debridement for infection being <14 days or ≥7 months from the definitive fixation surgery.

The clinical course of FRI is complex and challenges the sound methodologic evaluation of outcomes. For example, defining FRI vs aseptic nonunion is not always ascertainable. In addition, these patients often require multiple surgical procedures on the affected limb. Moreover, these patients often have multiple other injuries that may or may not require surgical treatment, but these injuries certainly affect the subsequent infection risk.

Overall, there was a large amount of variation in antibiotic treatment courses among and within the 4 institutions (Supplementary Table 2). The decision to treat for >6 vs ≤6 weeks was not clearly based on defined criteria or higher-risk features (eg, hardware removal). Some patients received oral stepdown therapy after a couple of weeks of intravenous antibiotics, while others received full courses of antibiotic therapy that was intravenous. Last, there was variable use of adjunctive rifampin in the treatment of staphylococcal FRI with retained hardware.

In this study, culture-negative FRI increased the hazard for infection- and surgery-free survival. This is supported by other studies that reported worse outcomes with culture-negative FRI, such as higher treatment failure, longer hospital stays, and higher amputation rate [16, 17]. The reason for this association is unknown but may be due to suboptimal antibiotic treatment. Furthermore, culture negativity at the time of debridement may indicate antibiotic treatment prior to culture collection, which may indicate a delay in debridement surgery that subsequently leads to worse outcomes. Culture negativity may also be due to the absence of infection; an anchoring bias on FRI as an explanation for delayed union, for example, may delay the treatment of important noninfectious causes. Along these lines, patients may be diagnosed with infection due to poor wound healing or other systemic illness, which leads to worse outcomes in general. FRI and prosthetic joint infection (PJI) are both serious complications following orthopedic surgery, yet the volume of medical literature reporting on these 2 types of infections is vastly different. According to a systematic review by Metsemakers et al, there were 10 029 publications on PJI and only 1740 publications on FRI from 2000 to 2015 [18]. PJI is more extensively studied and reported on than FRI, despite the fact that FRI is more common and has greater mortality rates than PJI [18]. The authors suggested that the discrepancy in the literature may be due to the lack of a clear definition and classification of FRI, difficulty in diagnosing and treating FRI, and decreased interest and awareness among clinicians and researchers with regard to FRI vs PJI. Therefore, there is a need for additional research and focus on FRIs to improve prevention and management of this challenging condition.

Our study does have some notable limitations. First, this was a retrospective cohort study. Antibiotic therapy duration was not randomly assigned and may have been based on specific patient characteristics. Although we were successful in weighting the analysis such that prognostic factors specified a priori were distributed evenly between the groups, unmeasured factors that influence physician decision as well as patient outcomes may bias our findings. Second, despite involvement of 4 academic centers and a 6-year study period, the study had a small number of patients, which limited our ability to address more specific questions based on pathogen type, antibiotic selection, or antibiotic duration other than the 6-week cutoff. Last, all 4 medical centers in the study were academic level 1 trauma centers, which may limit the generalizability of the results to other institutions and patient populations.

Despite these limitations, our study still provides preliminary evidence to support future research efforts to understand the optimal antibiotic treatment for FRI. Future considerations would include prospective or randomized trials to control for differential treatment selection bias. Additionally, we would advocate for the development of multicenter FRI databases to increase patient numbers and coordinate future research efforts. Last, a welcome objective for future studies would be to assess whether continuing antibiotic therapy until fracture consolidation results in better outcomes as compared with a fixed duration of antibiotic therapy when hardware is retained.

In conclusion, our US-based multicenter study on FRI found that a longer antibiotic course (>6 weeks) was not associated with higher surgery- or infection-free survival when compared with shorter antibiotic courses (≤6 weeks). This study provides additional data that can inform future studies investigating the ideal antibiotic treatment of FRI.

## Supplementary Material

ofae262_Supplementary_Data

## Supplementary Data

Supplementary materials are available at *Open Forum Infectious Diseases* online. Consisting of data provided by the authors to benefit the reader, the posted materials are not copyedited and are the sole responsibility of the authors, so questions or comments should be addressed to the corresponding author.

## Notes

***Author contributions.*** All authors have seen and approved the manuscript, contributed significantly to the work, and confirm that the manuscript has not been previously published and is not being considered for publication elsewhere.

***Patient consent statement.*** Our study does not include factors necessitating patient consent.

***Financial support.*** This work was supported by the Biostatistics, Epidemiology and Research Design Methods Core funded through grant UL1TR002553 from the National Center for Advancing Translational Sciences, a component of the National Institutes of Health.
