# Supplementary material for: Multisite Study of the Management of Musculoskeletal Infection After Trauma: The MMUSKIT Study
Source: Open Forum Infect Dis. 2024 May 6;11(6):ofae262. doi: 10.1093/ofid/ofae262 (PMC11161894; doi:10.1093/ofid/ofae262)
Supplement: ofae262_Supplementary_Data [file ofae262_supplementary_data.zip › Supplemental Fig 1.pdf]

Record Review

Record ID<br>{[record_id] text}	________________________________
Patient seen at:<br>{[hospital] radio Required}	◯ {1} Duke University<br>◯ {2} Massachusetts General Hospital<br>◯ {3} University of Florida<br>◯ {4} University of Utah
Patient medical record number<br>{[mrn] text Identifier}	________________________________<br>(this is only to help with importing records into redcap and will be removed prior to data download. Of note, in the rare instance where a patient has injuries in more than 1 extremity that go on to become infected, please enter a second record for the other limb if it also meets criteria for this study)
Patient Screening	
Should this patient be excluded from data review for any of the following criteria? Check as many as apply to this patient. If none are selected, please proceed with data entry.<br>{[screening] checkbox}	☐ {9} Patient did not have long bone trauma<br>☐ {1} Patient did not have an infection that was felt to involve the bone/hardware of the affected limb<br>☐ {2} Patient was less than 18 years of age at the time of ORIF<br>☐ {3} Less than one year has passed since debridement surgery<br>☐ {4} Patient received less than 2 weeks of antibiotics following debridement surgery<br>☐ {5} Debridement surgery was less than 14 days from definitive fixation surgery<br>☐ {6} Debridement surgery was more than 6 months following definitive fixation surgery<br>☐ {7} Patient was treated for infection at an outside institution.<br>☐ {8} Other
"Other" description<br>{[exclusion_other] text}<br>{Branching logic (show if): [screening(8)] = '1'}	________________________________

Original Injury

Date of original injury<br>{[injury_date] text (date_mdy) Required Identifier}	________________________________<br>(Date that the patient suferred their injury that resulted in a fracture on the limb that included them in the study)
Was the original injury a result of a crush injury<br>{[crush] yesno Required}	◯ Yes<br>◯ No<br>(as defined in the primary consultation note or H&P as a "crush" injury or "pinning")

Date of first surgery to address affected limb<br>{[first_surg] text (date_mdy) Identifier}	__________________________________
Was this first surgery done at an outside hospital?<br>{[initial_surg] yesno}	◯ Yes<br>◯ No
Days from original injury to first surgery<br>{[inj_to_first_surg] calc}	__________________________________
Date of definitive ORIF<br>{[orif_date] text (date_mdy) Required Identifier}	__________________________________<br>(Date of last surgery that the patient had during their initial trauma episode that relates to internal fixation of the infected limb)
Days from original injury to ORIF<br>{[inj_to_orif] calc}	__________________________________
Was the ORIF done at an outside hospital?<br>{[oif_osh] yesno}	◯ Yes<br>◯ No
Number of surgeries during initial trauma episode (on infected limb) including Ex-Fix, initial I&D for open fx, fasciotomies, etc.<br>{[surgeries_num] text (integer) Required}	__________________________________<br>(Count of the numbers of surgeries required during the initial trauma episode on the infected limb)
Was this a staged fixation?<br>{[staged_fixation] yesno Required}	◯ Yes<br>◯ No<br>(Did the initial repair of the infected limb require more than 1 surgery (as reflected in the prior question))
Was an external-fixator (ex-fix) used during staged procedure?<br>{[ex_fix] yesno}<br>{Branching logic (show if): [staged_fixation] = '1'}	◯ Yes<br>◯ No<br>(Was an external fixator applied as one of the surgeries referenced above)
Did the original injury result in a vascular injury?<br>{[vasc_injury_yn] yesno Required}	◯ Yes<br>◯ No
Did the original trauma result in a vascular injury that needed to be repaired?<br>{[vasc_injury] yesno Required}<br>{Branching logic (show if): [vasc_injury_yn] = '1'}	◯ Yes<br>◯ No<br>(Did the initial trauma result in damage to an artery or vein in the infected limb that required repair of the vessel?)
Did the original injury require a skin or soft tissue flap for repair?<br>{[flap] yesno Required}	◯ Yes<br>◯ No<br>(Did the initial injury require soft tissue mobilization (free or rotational) ?)

Which bone(s) was fractured in the limb that became infected?<br>{[bone] checkbox}	☐ {1} Tibia<br>☐ {2} Fibula<br>☐ {3} Humerus<br>☐ {4} Radius<br>☐ {5} Ulna<br>☐ {6} Femur<br>(Which bone(s) was fractured during the initial trauma in the limb that became infected?)
Did the fracture involve a joint space?<br>{[joint] yesno Required}	◯ Yes<br>◯ No<br>(Was the initial fracture in the infected limb continguous with a joint space?)
If so, which joint spaces were involved?<br>{[joint_name] checkbox}<br>{Branching logic (show if): [joint] = '1'}	☐ {1} Ankle<br>☐ {2} Knee<br>☐ {3} Hip<br>☐ {4} Wrist<br>☐ {5} Elbow<br>☐ {6} Shoulder
Was this injury part of a polytrauma?<br>{[polytrauma] yesno Required}	◯ Yes<br>◯ No<br>(Polytrauma is defined as two or more severe injuries in at least two areas of the body)
Was this an open or closed fracture?<br><br>https://www.uptodate.com/contents/image/print?imageKey=SURG%2F110486<br><br><br>{[open_closed] radio Required}	◯ {1} Closed, Comminuted (more than 2 fracture fragments)<br>◯ {2} Closed, Non-comminuted (2 fracture fragments)<br>◯ {3} Open, Gustilo Type I<br>◯ {4} Open, Gustilo Type 2<br>◯ {5} Open, Gustilo Type 3a<br>◯ {6} Open, Gustilo Type 3b<br>◯ {7} Open, Gustilo Type 3c<br>◯ {8} Closed, Other<br>◯ {9} Open, Other<br>(The link to the left provides open fracture classification definitions)
Was there gross environmental contamination at the time of the original injury?<br>{[gross_contam] radio Required}	◯ {1} Yes<br>◯ {2} No<br>◯ {3} Unknown<br>(Wound noted to have visible contamination of soil, feces, or water at the time of presentation)
Injury Severity Score<br>{[iss] text (integer Min: 3 Max: 75)}	______________________________<br>(this value should be available in original trauma note or trauma database. Values should be an integer and range from 3 to 75)
Hardware placed for definitive fixation of the infected limb<br>{[hardware] checkbox Required}	☐ {1} Plate and Screws<br>☐ {2} Intmaedullary Nail<br>☐ {3} Screws<br>☐ {4} Pins and/or K-wire<br>☐ {5} Other<br>(What kind of hardware was placed to repair the fracture of interest for this study?)

Please specify
{[hardware_other] text Required}
{Branching logic (show if): [hardware(5)] = '1'}

____

Perioperative antibiotics administered for the definitive fixation surgery
{[abx_fixation] checkbox Required}

- ☐ {1} Aztreonam
- ☐ {2} Cefazolin
- ☐ {3} Ceftriaxone
- ☐ {4} Cefepime
- ☐ {5} Ciprofloxacin
- ☐ {6} Levofloxacin
- ☐ {7} Clindamycin
- ☐ {8} Gentamicin
- ☐ {9} Metronidazole
- ☐ {10} Vancomycin
- ☐ {15} Penicillin
- ☐ {11} Piperacillin-Tazobactam
- ☐ {12} Other
- ☐ {13} None
- ☐ {14} Unknown

(Which antibiotics were administered perioperatively for the definitive fixation surgery for the original injury?)

Name of the perioperative antibiotic
{[abx_fix_other] text}
{Branching logic (show if): [abx_fixation(12)] = '1'}

____

Were there local antibiotics placed into the surgical site during the definitive fixation surgery?
{[local_abx_fix] checkbox Required}

- ☐ {1} Vancomycin
- ☐ {2} Tobramycin
- ☐ {3} Ceftazidime
- ☐ {4} Gentamicin
- ☐ {5} Other
- ☐ {6} None
- ☐ {7} Unknown

(Which antibiotic was placed locally at the site of the fracture at the time of the definitive fixation surgery?)

Name of the local antibiotic used
{[local_abx_fix_other] text}
{Branching logic (show if): [local_abx_fix(5)] = '1'}

____

What was the format of the local antibiotics used during the definitive fixation surgery?
{[local_abx_fix_format] checkbox Required}

- ☐ {1} Powder
- ☐ {2} PMMA cement nail or spacer
- ☐ {3} PMMA cement beads
- ☐ {4} Calcium sulfate beads
- ☐ {5} No local antibiotics used

Initial Debridement for Infection

Date of initial debridement surgery for infection
{[infx_surg_date] text (date_mdy) Required Identifier}

____
(Date that the patient underwent surgery to revise or debride hardware in place from the definitive fixation surgery. Of note, this surgery should only be included in the study if the infection occurred after the patient was done with their fixation surgeries, as opposed to if the infection occurred while definitive surgery was still not complete.)

Days from ORIF to debridement surgery<br>{[orif_to_debride] calc}	______________________
Did the infection surgery occur during the same hospitalization as the definitive fixaiton surgery?<br>{[same_hosp] yesno Required}	◯ Yes<br>◯ No
Was the patient more than 89 years of age at the time of the first infection surgery?<br>{[gt_89] yesno}	◯ Yes<br>◯ No
Age of patient at the time of the first infection surgery<br>{[age] text Required Identifier}<br>{Branching logic (show if): [gt_89] = '0'}	______________________<br>(How many years old was the patient at the time of the first infection surgery?)
Body mass index (BMI) of the patient at the time of the intial infection surgery<br>{[bmi] text Required}	______________________<br>(BMI= kg/m2)
Sex of the patient<br>{[sex] radio Required}	◯ {1} Male<br>◯ {2} Female<br>◯ {3} Other<br>(Male, Female or Other based on genitalia and chromosome composition)
Gender<br>{[gender] radio}	◯ {1} Woman<br>◯ {2} Man<br>◯ {3} Transgender<br>◯ {4} Non-binary/non-conforming<br>◯ {5} Unknown
Did the patient have a diagnosis of diabetes mellitus at the time of initial infection surgery?<br>{[diabetes] yesno Required}	◯ Yes<br>◯ No<br>(Is the patient on medication to prevent hyperglycemia or have a hemoglobin A1c equal to or greater than 6.5%)
American Society of Anesthesiology (ASA) score for the patient at the time of the initial infection surgery<br>{[asa] radio}	◯ {1} Grade 1 (normal healthy patient)<br>◯ {2} Grade II (patient with mild systemic disease)<br>◯ {3} Grade III (patient with severe systemic disease)<br>◯ {4} Grade IV (patient with severe systemic disease that is constant threat to life)<br>◯ {5} Grade V (moribund patient who is not expected to survive without the operation)<br>◯ {6} Grade VI (declared brain-dead patient whose organs are being removed for donor purposes)<br>◯ {7} Unknown<br>(Should be defined in anesthesia record)

Smoking status of patient at the time of initial debridement surgery?<br>{[smoker] radio Required}	○ {1} Current smoker (has smoked 100 cigarettes in his or her lifetime and who currently smokes cigarettes)<br>○ {2} Prior smoker (smoked at least 100 cigarettes in his or her lifetime but who now abstains from smoking cigarettes)<br>○ {3} Never smoker (has smoked less than 100 cigarettes in his or her lifetime and currently abstains from smoking cigarettes)<br>○ {4} Unknown
Was the patient immunosuppressed at the time of the initial infection surgery?<br>{[immunosuppressed] checkbox Required}	☐ {1} Steroids (>20mg daily for > 4 weeks)<br>☐ {2} Biologic medication<br>☐ {3} Solid organ transplant recipient<br>☐ {4} HIV infection (CD4< 200)<br>☐ {5} Hematologic malignancy<br>☐ {6} Solid organ cancer on chemotherapy (has received chemotherapy in the 6 months prior to infection surgery)<br>☐ {7} Bone marrow transplant recipient<br>☐ {8} Not immunosuppressed<br>☐ {9} Unknown
Was the patient receiving hemodialysis at the time of the initial infection surgery?<br>{[hd] radio Required}	○ {1} Yes<br>○ {2} No<br>○ {3} Unknown
Patient's C-reactive protein (CRP) level recorded 2 weeks prior to or 2 weeks following initial infection surgery. If multiple values exist please pick the value closest to the date of surgery. If no value is available, please leave this answer blank.<br>{[crp] text}	____________________<br>(mg/dL)
Patient's sedimentation rate (ESR) level recorded 2 weeks prior to or 2 weeks following initial infection surgery. If multiple values exist please pick the value closest to the date of surgery. If no value is available, please leave this answer blank<br>{[esr] text}	____________________<br>(mm/hr)
Patient's white blood cell count (WBC) recorded 2 weeks prior to or 2 weeks following initial infection surgery. If multiple values exist please pick the value closest to the date of surgery. If no value is available, please leave this answer blank.<br>{[wbc] text}	____________________<br>(10^9/L)
Patient's albumin level recorded 2 weeks prior to or 2 weeks following initial infection surgery. If multiple values exist please pick the value closest to the date of surgery. If no value is available, please leave this answer blank.<br>{[albumin] text}	____________________<br>(g/dL)

Number of surgeries required to manage 1st infection episode<br>{[infxn_surg_num] text (integer Min: 1 Max: 999) Required}	______________________________<br>(How many surgeries did the patient require to manage the first episode of infection on the affected limb (including the initial infection surgery)? This only includes debridement in the operating room)
Pathogens isolated from infection surgery<br>{[organism] checkbox}	☐ {1} MRSA<br>☐ {2} MSSA<br>☐ {3} Staphylococcus lugdunensis<br>☐ {4} Coagulase-negative staphylococcus (excludes Staphylococcus lug)<br>☐ {5} E. coli<br>☐ {6} Klebsiella species<br>☐ {7} Enterobacter spp<br>☐ {8} Enterococcus spp<br>☐ {9} Streptococcus spp<br>☐ {10} Clostridium spp<br>☐ {11} Proteus spp<br>☐ {12} Serratia spp<br>☐ {13} Pseudomonas spp<br>☐ {14} Bacteroides spp<br>☐ {15} Cutibacterium spp<br>☐ {16} Other aerobic gram positive cocci<br>☐ {17} Other aerobic gram negative rod/bacilli<br>☐ {18} Mold<br>☐ {19} Other/mixed anaerobe(s)<br>☐ {20} Candida spp<br>☐ {21} Non-tuberculus mycobacteria (NTM)<br>☐ {22} Culture-negative<br>☐ {23} No culture taken<br>(Which pathogen(s) was isolated during any of the infection surgeries to manage the first infection episode. Only includes cultures taken under sterile conditions and excludes bedside debridements)
Please specify other aerobic gram positive cocci<br>{[organism_other_aero_gpc] text}<br>{Branching logic (show if): [organism(16)] = '1'}	______________________________
Please specify other aerobic gram negative rod/bacilli<br><br>{[organism_other_aero_gnb] text}<br>{Branching logic (show if): [organism(17)] = '1'}	______________________________
Please specify mold<br>{[organism_other_aero_gnr] text}<br>{Branching logic (show if): [organism(18)] = '1'}	______________________________
Please specify other mixed/anaerobes<br>{[organism_other_mixed] text}<br>{Branching logic (show if): [organism(19)] = '1'}	______________________________

Were any of the pathogens listed above considered resistant organisms?

Reistant Organism Definition
MRSA

Includes Staphylococcus aureus cultured from any specimen that tests oxacillin-resistant, cefoxitin-resistant, or methicillin-resistant by standard susceptibility testing methods, or by a laboratory test that is FDA-approved for MRSA detection from isolated colonies; these methods may also include a positive result by any FDA approved test for MRSA detection from specific sources.

VRE

Any Enterococcus spp. (regardless of whether identified to the species level), that is resistant to vancomycin, by standard susceptibility testing methods or by results from any FDA-approved test for VRE detection from specific specimen sources.

CRE Any Enterobacteriaceae spp. testing resistant to any carbapenem including doripenem, ertapenem, imipenem or meropenem using the current CLSI breakpoints; or by a positive result for any method FDA approved for carbapenemase detection.

ESBL -Enterobacteriaceae spp. non-susceptible (i.e., resistant or intermediate) to ceftazidime, cefepime, ceftriaxone, or cefotaxime.

-Pseudomonas aeruginosa non-susceptible (i.e., resistant or intermediate) to ceftazidime or cefepime.

Multi-drug resistant gram negative organism Non-susceptibility (i.e., resistant or intermediate) to at least one agent in at least 3 antimicrobial classes of the following 6 classes:

-Ampicillin/sulbactam

-Cephalosporins (cefepime, ceftazidime)

-β-lactam/β-lactam β-lactamase inhibitor combination (piperacillin, piperacillin/tazobactam)

-Carbapenems (imipenem, meropenem, doripenem)

-Fluoroquinolones (ciprofloxacin or levofloxacin)

-Aminoglycosides (gentamicin, tobramycin, or amikacin)

{[resistant_orgs] checkbox Required}

☐ {1} methicillin resistant staph aureus (MRSA)
☐ {2} vancomycin resistant enterococcus (VRE)
☐ {3} multi-drug resistant organism (MDRO)
☐ {4} extended-spectrum beta lactamase (ESBL)
☐ {5} carbapenem resistant enterobacteriacae (CRE)
☐ {6} none of the above

Which topical antibiotics were used during the surgeries to treat the initial infection?<br>{[local_abx_inf] checkbox Required}	☐ {1} Vancomycin<br>☐ {2} Tobramycin<br>☐ {3} Ceftazidime<br>☐ {4} Gentamicin<br>☐ {5} Other<br>☐ {6} None<br>☐ {7} Unknown
Please Specify<br>{[local_abx_inf_other] text Required}<br>{Branching logic (show if): [local_abx_inf(5)] = '1'}	________________________
What was the format of the local antibiotics used during the infection surgery/surgeries?<br>{[local_abx_inf_format] checkbox Required}	☐ {1} Powder<br>☐ {2} PMMA cement nail or spacer<br>☐ {3} PMMA cement beads<br>☐ {4} Calcium sulfate beads<br>☐ {5} No local antibiotics used
During any of the surgeries to treat the initial infection, was all of the hardware removed? None of the hardware removed? Or some of the hardware removed?<br><br>{[hardware_removed] radio}	◯ {1} All hardware removed<br>◯ {2} No hardware removed<br>◯ {3} Partial hardware removal<br>(this only includes hardware in the infected limb)
During any of the surgeries to treat the initial infection, was new hardware placed or replaced?<br>{[hardware_replaced] radio Required}	◯ {1} Yes<br>◯ {2} No<br>◯ {3} Unsure
Was infectious diseases consulted in order ot help treat this trauma-ID infection?<br>{[id_consult] yesno}	◯ Yes<br>◯ No

Which systemic antibiotics were used during the first 6 weeks to treat the infection? This will be referred to as the "induction therapy" in subsequent questions
{[abx_induction] checkbox}

- ☐ {36} Amikacin
- ☐ {1} Amoxicillin
- ☐ {2} Amoxicillin-clavulanate
- ☐ {3} Ampicillin
- ☐ {4} Ampicillin-sulbactam
- ☐ {5} Cefazolin
- ☐ {6} Cefepime
- ☐ {7} Cefiderocol
- ☐ {8} Ceftaroline
- ☐ {9} Ceftazidime
- ☐ {10} Ceftazidime-avibactam
- ☐ {11} Ceftolozone/tazobactam
- ☐ {12} Ceftriaxone
- ☐ {37} Cefuroxime
- ☐ {13} Ciprofloxacin
- ☐ {14} Clindamycin
- ☐ {15} Dalbavancin
- ☐ {16} Daptomycin
- ☐ {17} Doxycycline
- ☐ {18} Ertapenem
- ☐ {19} Gentamicin
- ☐ {20} Imipenem
- ☐ {21} Levofloxacin
- ☐ {22} Linezolid
- ☐ {23} Meropenem
- ☐ {24} Metronidazole
- ☐ {38} Minocycline
- ☐ {25} Nafcillin
- ☐ {26} Oritavancin
- ☐ {27} Oxacillin
- ☐ {28} Penicillin G
- ☐ {29} Pipercillin/Tazobactam
- ☐ {30} Tedizolid
- ☐ {31} Tobramycin
- ☐ {32} Trimethoprim-sulfamethoxazole
- ☐ {33} Vancomycin
- ☐ {34} Other
- ☐ {35} None

(Only include antibiotics that were given for at least 2 weeks)

Please specify other antibiotics used:
{[abx_induction_other] text Required}
{Branching logic (show if): [abx_induction(34)] = '1'}

____
(if multiple, seperate with semi-colon)

How many weeks of antibiotics did the patient receive for "induction" therapy (e.g. cumulative sum of all of the antibiotics checked above)
{[induction_end_date] radio}

- ◯ {1} 2.0-2.6 weeks
- ◯ {2} 3-3.6 weeks
- ◯ {3} 4-4.6 weeks
- ◯ {4} 5-5.6 weeks
- ◯ {5} 6-6.6 weeks
- ◯ {6} 7-7.6 weeks
- ◯ {7} 8-8.6 weeks
- ◯ {8} 9-9.6 weeks
- ◯ {9} 10-10.6 weeks
- ◯ {10} 11-11.6 weeks
- ◯ {11} 12-12.6 weeks
- ◯ {12} > 12.6 weeks

Estimated number of days of induction therapy
{[est_ind_end_12] text (integer Min: 92 Max: 500)}
{Branching logic (show if): [induction_end_date] = '12'}

____

{[est_ind_days] calc}

____

{[est_ind_days_cum] calc}

____

Was adjunctive rifampin used to treat the initial infection while the patient was on "induction therapy"?
{[rifampin_induction] radio}

- ◯ {1} Yes
- ◯ {2} No
- ◯ {3} Unsure

(rifampin must be used for at least 14 days of treatment in order to qualify)

During what week of induction therapy was rifampin added?
{[week_rif_add] radio}
{Branching logic (show if): [rifampin_induction] = '1'}

- ◯ {1} 0-0.6
- ◯ {2} 1-1.6
- ◯ {3} 2-2.6
- ◯ {4} 3-3.6
- ◯ {5} 4-4.6
- ◯ {6} 5-5.6

Were oral antibiotics used for maintenance therapy (e.g. following end date listed above for "induction therapy")? If so, were the antibiotics intended to be used indefinitely or until the fracture healed?
{[oral_abx_duration] radio}

- ◯ {1} Indefinitely
- ◯ {2} Until the fracture healed
- ◯ {3} No oral antibiotics used
- ◯ {4} Unclear duration
- ◯ {5} Defined duration (e.g. 6 weeks of maintenance therapy)

How many weeks was the patient on maintenance therapy?

{[maintenance_dur] text (integer Min: 1 Max: 999)}
{Branching logic (show if): [oral_abx_duration] = '5'}

____

{[days_main_tx_calc] calc}

____

{[abx_days_total] calc}

____

Which oral antibiotics were prescribed for "maintenance therapy" of the infection? (e.g. following induction)
{[oral_abx_name] checkbox}
{Branching logic (show if): [oral_abx_duration] = '1' or [oral_abx_duration] = '2' or [oral_abx_duration] = '4' or [oral_abx_duration] = '5'}

- ☐ {1} Cefadroxil
- ☐ {2} Cephalexin
- ☐ {3} Dicloxacillin
- ☐ {4} Trimethoprim-sulfamethoxazole
- ☐ {5} Doxycycline
- ☐ {6} Minocycline
- ☐ {7} Clindamycin
- ☐ {8} Ciprofloxacin
- ☐ {9} Levofloxacin
- ☐ {10} Amoxicillin
- ☐ {11} Amoxicillin-clavulanate
- ☐ {12} Penicillin VK
- ☐ {15} Linezolid
- ☐ {16} Cefuroxime
- ☐ {17} Cefpodoxime
- ☐ {13} Other
- ☐ {14} None

What was the name of the oral antibiotic?
{[oral_abx_other] text}
{Branching logic (show if): [oral_abx_name(13)] = '1'}

____

Was rifampin used as adjunctive therapy during maintenance therapy (e.g. following the induction treatment)?<br>{[rifampin_maintenance] radio}	◯ {1} Yes<br>◯ {2} No<br>◯ {3} Unsure<br>(must be used for at least 14 days in order to qualify)
For how many total weeks was rifampin used as adjunctive therapy? (induction + maintenance)<br>{[rifampin_weeks] text (integer Min: 1 Max: 999)}<br>{Branching logic (show if): [rifampin_maintenance] = '1' or [rifampin_induction] = '1'}	______________________

Outcomes

Did the patient return to the OR more than 2 weeks after the last planned infection surgery to treat the first infection?<br>{[or_return] yesno Required}	◯ Yes<br>◯ No<br>(This only includes surgery on the limb that required debridement to treat the first episode of infection. Again, it should be at least 2 weeks following the last planned surgical intervention to treat the infection)
How many times did the patient return to the OR more than 2 weeks after the last planned infection surgery to treat the first infection?<br>{[or_return_surgery_number] checkbox}<br>{Branching logic (show if): [or_return] = '1'}	☐ {1} 1<br>☐ {2} 2<br>☐ {3} 3<br>☐ {4} 4<br>☐ {5} 5<br>☐ {6} 6
First date that the patient returned to the OR in the question above<br>{[or_return_date] text (date_mdy) Identifier}<br>{Branching logic (show if): [or_return] = '1'}	______________________
Second date that the patient returned to the OR in the question above<br>{[or_return_date_2] text (date_mdy) Identifier}<br>{Branching logic (show if):<br>[or_return_surgery_number(2)] = '1' or<br>[or_return_surgery_number(3)] = '1' or<br>[or_return_surgery_number(4)] = '1' or<br>[or_return_surgery_number(5)] = '1' or<br>[or_return_surgery_number(6)] = '1'}	______________________
Third date that the patient returned to the OR in the question above<br>{[or_return_date_3] text (date_mdy) Identifier}<br>{Branching logic (show if):<br>[or_return_surgery_number(3)] = '1' or<br>[or_return_surgery_number(4)] = '1' or<br>[or_return_surgery_number(5)] = '1' or<br>[or_return_surgery_number(6)] = '1'}	______________________

Fourth date that the patient returned to the OR in the question above
{[or_return_date_4] text (date_mdy) Identifier}
{Branching logic (show if):
[or_return_surgery_number(4)] = '1' or
[or_return_surgery_number(5)] = '1' or
[or_return_surgery_number(6)] = '1'}

____

Fifth date that the patient returned to the OR in the question above
{[or_return_date_5] text (date_mdy) Identifier}
{Branching logic (show if):
[or_return_surgery_number(5)] = '1' or
[or_return_surgery_number(6)] = '1'}

____

Sixth date that the patient returned to the OR in the question above
{[or_return_date_6] text (date_mdy) Identifier}
{Branching logic (show if):
[or_return_surgery_number(6)] = '1'}

____

Days from initial debridement surgery to subsequent surgery
{[debride_to_second_or] calc}
{Branching logic (show if): [or_return] = '1'}

____

Days from initial debridement surgery to 2nd return to the OR noted above
{[debride_to_second_or_2] calc}
{Branching logic (show if):
[or_return_surgery_number(2)] = '1' or
[or_return_surgery_number(3)] = '1' or
[or_return_surgery_number(4)] = '1' or
[or_return_surgery_number(5)] = '1' or
[or_return_surgery_number(6)] = '1'}

____

Days from initial debridement surgery to 3rd return to the OR noted above
{[debride_to_second_or_3] calc}
{Branching logic (show if):
[or_return_surgery_number(3)] = '1' or
[or_return_surgery_number(4)] = '1' or
[or_return_surgery_number(5)] = '1' or
[or_return_surgery_number(6)] = '1'}

____

Days from initial debridement surgery to 4th return to the OR noted above
{[debride_to_second_or_4] calc}
{Branching logic (show if):
[or_return_surgery_number(4)] = '1' or
[or_return_surgery_number(5)] = '1' or
[or_return_surgery_number(6)] = '1'}

____

Days from initial debridement surgery to 5th return to the OR noted above
{[debride_to_second_or_5] calc}
{Branching logic (show if):
[or_return_surgery_number(5)] = '1' or
[or_return_surgery_number(6)] = '1'}

____

Days from initial debridement surgery to 6th return to the OR noted above<br>{[debride_to_second_or_6] calc}<br>{Branching logic (show if): [or_return_surgery_number(6)] = '1'}	______________________________
Was the patient still on antibiotic therapy on the date(s) of the return to the OR listed above?<br>{[abx_return_surg] yesno}<br>{Branching logic (show if): [or_return] = '1'}	◯ Yes<br>◯ No<br>(click "yes" if the patient was on antibiotics during any of the subsequent operations following debridement surgery)
What was the indication for the return to the OR on the previously infected limb?<br>{[or_return_ind] checkbox}<br>{Branching logic (show if): [or_return] = '1'}	☐ {1} Concern for infection<br>☐ {2} Hardware failure<br>☐ {3} Repeat fracture/trauma<br>☐ {4} Painful hardware<br>☐ {5} Non-union<br>☐ {6} Need for soft tissue coverage<br>☐ {7} Other<br>☐ {8} Unclear
What was the reason that the patient returned to the OR?<br>{[return_ind_other] text}<br>{Branching logic (show if): [or_return_ind(7)] = '1'}	______________________________
Was the return to the OR planned or unplanned?<br>{[planned_return_or] checkbox}<br>{Branching logic (show if): [or_return] = '1'}	☐ {1} Planned<br>☐ {2} Unplanned<br>☐ {3} Not specified<br>(If the surgeon documented a need for the patient to go back to the OR prior to presentation to an ED/Hospital visit then would consider this to be a planne revision/debridement. If the patient presented for an acute issue to an ED/Hospital and the decision was made to bring them back to the OR, then would consider this unplanned)
Was the pathogen(s) found during the repeat surgery the same as the pathogen(s) during the initial infection surgery/surgeries?<br>{[repeat_or_pathogen] checkbox}<br>{Branching logic (show if): [or_return] = '1'}	☐ {1} Same pathogen(s)<br>☐ {2} New pathogen(s)<br>☐ {3} Negative culture<br>☐ {4} No cultures taken
At the time of repeat surgery, was there radiographic evdience of infection?<br>{[radiology_infxn] checkbox}<br>{Branching logic (show if): [abx_return_surg] = '1'}	☐ {1} Hardware loosening<br>☐ {2} Osteomyelitis<br>☐ {3} Abscess<br>☐ {4} No evidence of infection on available imaging<br>☐ {5} No imaging ordered during this time<br>(radiology should be performed as workup prior to return to the OR, but following the last infection surgery)
During any the repeat operations, was there concern for infection?<br>{[or_infxn_concern] radio}<br>{Branching logic (show if): [or_return] = '1'}	◯ {1} Yes<br>◯ {2} No<br>◯ {3} Unsure<br>(based on intraoperative observation)
After the surgery at which infection was identified, were there additional surgeries to promote union??<br>{[union_surg] yesno}	◯ Yes<br>◯ No<br>(e.g. bone grafting)

Does the most recent radiology study of the affected limb show evidence of union?<br>{[recent_rad] radio}	◯ {1} Yes<br>◯ {2} No<br>◯ {3} No recent radiology available<br>(Based on documentation from physicial interpretation or official radiology interpretation)
Date of most recent radiology study<br>{[rad_date] text (date_mdy) Identifier}<br>{Branching logic (show if): [recent_rad] = '1' or [recent_rad] = '2'}	______________________
Days from original surgery to treat infection (e.g. original debridement surgery) to most recent radiology study<br>{[debride_to_rad] calc}<br>{Branching logic (show if): [recent_rad] = '1' or [recent_rad] = '2'}	______________________
Date of last follow up note from ID or Orthopedics<br>{[id_ortho_f_u] text (date_mdy) Identifier}	______________________
Days from original debridement to last ID or Orthopedics Follow up<br>{[debride_to_fu] calc}	______________________
At the time of last follow up in ID or Orthopedics clinics was there any concern for ongoing infection?<br>{[infx_concern] yesno}	◯ Yes<br>◯ No<br>(as indicated in visit note)
Date of most recent follow up visit in the electronic medical record that documents symptoms and/or physical exam of the previously infected limb<br>{[last_follow_up] text (date_mdy) Required Identifier}	______________________
Days from debridement to last EMR documentation<br>{[debride_to_last_doc] calc}	______________________
At the time of this last follow up in the electronic medical record, is the patient on antibiotics to treat the infected limb?<br>{[abx_follow_up] yesno Required}	◯ Yes<br>◯ No
At the time of the last follow up in the electronic medical record, did the patient undergo an amputation on the infected limb?<br>{[amputation] yesno}	◯ Yes<br>◯ No
Does the electronic medical record indicate that the patient has died?<br>{[death] yesno Required}	◯ Yes<br>◯ No
Date of amputation. If not known leave blank<br>{[amp_date] text (date_mdy) Identifier}<br>{Branching logic (show if): [amputation] = '1'}	______________________

Date of death recorded in the electronic medical record. If unknown, leave blank<br>{[death_date] text (date_mdy) Identifier}<br>{Branching logic (show if): [death] = '1'}	______________________
Days from initial surgery to amputation<br>{[amp_days] calc}	______________________
Days from initial debridement surgery to death<br>{[debride_to_death] calc}<br>{Branching logic (show if): [death] = '1'}	______________________
Cause of death related to infection?<br>{[cause_of_death] radio}<br>{Branching logic (show if): [death] = '1'}	◯ {1} Definitely related<br>◯ {2} Likely related<br>◯ {3} Possibly related<br>◯ {4} Not related<br>◯ {5} Unable to determine<br>◯ {6} Unknown
Did the patient achieve union?<br>{[union] dropdown}	◯ {1} Yes<br>◯ {2} No<br>◯ {3} Unknown<br>(Based on radiology report or surgeon documentation)
Was there one year of followup in the EMR after antibiotics were stopped?<br>{[year_follow_up] yesno}	◯ Yes<br>◯ No
Was there any evidence of recurrent infection after antibiotics were stopped? (E.g. was the patient treated with antibiotics for a bone infection on the affected limb follwing antibiotic treatment for the the first infeciton episode?)<br>{[recurrent_infxn] yesno}	◯ Yes<br>◯ No
