# Supplementary material for: Multisite Study of the Management of Musculoskeletal Infection After Trauma: The MMUSKIT Study
Source: Open Forum Infect Dis. 2024 May 6;11(6):ofae262. doi: 10.1093/ofid/ofae262 (PMC11161894; doi:10.1093/ofid/ofae262)
Supplement: ofae262_Supplementary_Data [file ofae262_supplementary_data.zip › Supplementary Table Legends.docx]

Supplemental Table 1

| **Characteristics of Index operation** | | | |
| --- | --- | --- | --- |
|  | ≤ 6Weeks (N=54) | >6 Weeks (N=42) | Total (N=96) |
| **Days from original injury to first surgery** |  |  |  |
| N | 53 | 41 | 94 |
| Mean (SD) | 3.7 (16.1) | 1.9 (3.3) | 2.9 (12.2) |
| Median (Q1, Q3) | 1.0 (1.0, 1.0) | 1.0 (0.0, 1.0) | 1.0 (0.0, 1.0) |
| (Min, Max) | (0.0, 117.0) | (0.0, 17.0) | (0.0, 117.0) |
| **Days from original injury to ORIF** |  |  |  |
| N | 54 | 42 | 96 |
| Median (Q1, Q3) | 3.0 (1.0, 14.0) | 4.0 (1.0, 8.0) | 3.0 (1.0, 11.5) |
| (Min, Max) | (0.0, 117.0) | (0.0, 25.0) | (0.0, 117.0) |
| **Number of surgeries during initial trauma episode** |  |  |  |
| **(on infected limb)** |  |  |  |
| N | 53 | 42 | 95 |
| Median (Q1, Q3) | 1.0 (1.0, 3.0) | 1.0 (1.0, 2.0) | 1.0 (1.0, 3.0) |
| (Min, Max) | (1.0, 8.0) | (1.0, 9.0) | (1.0, 9.0) |
| **External-fixator used during staged procedure** |  |  |  |
| Not a staged procedure | 27 | 22 | 49 |
| No | 2 (7.4%) | 1 (5.0%) | 3 (6.4%) |
| Yes | 25 (92.6%) | 19 (95.0%) | 44 (93.6%) |
| **Skin or soft tissue flap required** | 7 (13.0%) | 7 (16.7%) | 14 (14.6%) |
| **Which bone(s) was fractured in the limb that became infected?** |  |  |  |
| Tibia | 46 (85.2%) | 35 (83.3%) | 81 (84.4%) |
| Fibula | 28 (51.9%) | 28 (66.7%) | 56 (58.3%) |
| Radius | 1 (1.9%) | 0 (0.0%) | 1 (1.0%) |
| Ulna | 1 (1.9%) | 0 (0.0%) | 1 (1.0%) |
| Femur | 10 (18.5%) | 8 (19.0%) | 18 (18.8%) |
| **Open or Closed fracture?** |  |  |  |
| Closed, Comminuted (more than 2 fracture | 21 (38.9%) | 17 (40.5%) | 38 (39.6%) |
| fragments) |  |  |  |
| Closed, Non-comminuted (2 fracture fragments) | 8 (14.8%) | 2 (4.8%) | 10 (10.4%) |
| Open, Gustilo Type I | 3 (5.6%) | 0 (0.0%) | 3 (3.1%) |
| Open, Gustilo Type 2 | 6 (11.1%) | 7 (16.7%) | 13 (13.5%) |
| Open, Gustilo Type 3a | 6 (11.1%) | 9 (21.4%) | 15 (15.6%) |
| Open, Gustilo Type 3b | 2 (3.7%) | 3 (7.1%) | 5 (5.2%) |
| Open, Gustilo Type 3c | 1 (1.9%) | 2 (4.8%) | 3 (3.1%) |
| Closed, Other | 2 (3.7%) | 1 (2.4%) | 3 (3.1%) |
| Open, Other | 5 (9.3%) | 1 (2.4%) | 6 (6.3%) |
| **Gross environmental contamination** |  |  |  |
| Yes | 12 (22.2%) | 10 (23.8%) | 22 (22.9%) |
| No | 39 (72.2%) | 27 (64.3%) | 66 (68.8%) |
| Unknown | 3 (5.6%) | 5 (11.9%) | 8 (8.3%) |
| **Hardware placed for definitive fixation of the infected limb** |  |  |  |
| Plate and screws | 35 (64.8%) | 31 (73.8%) | 66 (68.8%) |
| Intramedullary nail | 19 (35.2%) | 11 (26.2%) | 30 (31.3%) |
| Screws | 17 (31.5%) | 7 (16.7%) | 24 (25.0%) |
| Pins and/or K-wire | 3 (5.6%) | 3 (7.1%) | 6 (6.3%) |
| Other | 2 (3.7%) | 3 (7.1%) | 5 (5.2%) |
| **Local antibiotics during definitive fixation surgery** | 15 (27.8%) | 22 (52.4%) | 37 (38.5%) |
| Vancomycin | 13 (24.1%) | 20 (47.6%) | 33 (34.4%) |
| Tobramycin | 8 (14.8%) | 5 (11.9%) | 13 (13.5%) |
| Ceftazidime | 0 (0.0%) | 7 (16.7%) | 7 (7.3%) |
| Gentamicin | 1 (1.9%) | 3 (7.1%) | 4 (4.2%) |
| Other | 1 (1.9%) | 1 (2.4%) | 2 (2.1%) |
| Unknown | 4 (7.4%) | 1 (2.4%) | 5 (5.2%) |
| **Format of local antibiotics during fixation surgery** |  |  |  |
| Powder | 14 (25.9%) | 15 (35.7%) | 29 (30.2%) |
| PMMA cement nail or spacer | 1 (1.9%) | 5 (11.9%) | 6 (6.3%) |
| PMMA cement beads | 0 (0.0%) | 1 (2.4%) | 1 (1.0%) |
| Calcium sulfate beads | 0 (0.0%) | 1 (2.4%) | 1 (1.0%) |

Supplemental Table 2

|  | | | | | |
| --- | --- | --- | --- | --- | --- |
|  | Duke University (N=25) | Massachusetts General Hospital (N=17) | University of Florida (N=20) | University of Utah (N=34) | Total (N=96) |
| **Age (Years)** |  |  |  |  |  |
| N | 25 | 17 | 20 | 34 | 96 |
| Mean (SD) | 48.5 (17.3) | 62.2 (16.8) | 49.0 (15.9) | 45.0 (17.1) | 49.8 (17.7) |
| Median (Q1, Q3) | 48.0 (38.0, 58.0) | 61.0 (56.0, 75.0) | 49.0 (34.5, 59.5) | 43.0 (33.0, 54.0) | 49.0 (34.5, 60.0) |
| (Min, Max) | (18.0, 81.0) | (28.0, 89.0) | (23.0, 77.0) | (18.0, 87.0) | (18.0, 89.0) |
| **Male** | 17 (68.0%) | 9 (52.9%) | 9 (45.0%) | 21 (61.8%) | 56 (58.3%) |
| **ASA score** |  |  |  |  |  |
| Missing | 0 (.%) | 0 (.%) | 2 (.%) | 0 (.%) | 2 |
| Grade 1 (normal healthy patient) | 1 (4.0%) | 1 (5.9%) | 1 (5.6%) | 6 (17.6%) | 9 (9.6%) |
| Grade II (patient with mild systemic disease) | 12 (48.0%) | 11 (64.7%) | 6 (33.3%) | 12 (35.3%) | 41 (43.6%) |
| Grade III (patient with severe systemic | 12 (48.0%) | 5 (29.4%) | 11 (61.1%) | 14 (41.2%) | 42 (44.7%) |
| disease) |  |  |  |  |  |
| Grade IV (patient with severe systemic | 0 (0.0%) | 0 (0.0%) | 0 (0.0%) | 2 (5.9%) | 2 (2.1%) |
| disease that is constant threat to life) |  |  |  |  |  |
| **Fractured bone** |  |  |  |  |  |
| Tibia | 22 (88.0%) | 17 (100.0%) | 15 (75.0%) | 27 (79.4%) | 81 (84.4%) |
| Fibula | 9 (36.0%) | 8 (47.1%) | 13 (65.0%) | 26 (76.5%) | 56 (58.3%) |
| Radius | 0 (0.0%) | 0 (0.0%) | 0 (0.0%) | 1 (2.9%) | 1 (1.0%) |
| Ulna | 0 (0.0%) | 0 (0.0%) | 0 (0.0%) | 1 (2.9%) | 1 (1.0%) |
| Femur | 6 (24.0%) | 1 (5.9%) | 4 (20.0%) | 7 (20.6%) | 18 (18.8%) |
| **Antibiotic weeks** |  |  |  |  |  |
| N | 25 | 17 | 20 | 34 | 96 |
| Mean (SD) | 10.7 (7.4) | 12.4 (13.0) | 10.5 (5.2) | 9.9 (5.2) | 10.7 (7.6) |
| (Min, Max) | (4.9, 36.9) | (4.9, 52.9) | (2.9, 18.9) | (2.9, 26.9) | (2.9, 52.9) |
| **Days from debridement to last EMR documentation** |  |  |  |  |  |
| N | 25 | 17 | 20 | 34 | 96 |
| Median (Q1, Q3) | 763.0 (433.0, 1636.0) | 697.0 (241.0, 1003.0) | 377.0 (185.5, 803.5) | 368.0 (185.0, 1213.0) | 490.0 (229.5, 1226.0) |
| (Min, Max) | (197.0, 2998.0) | (68.0, 1770.0) | (43.0, 2283.0) | (75.0, 2744.0) | (43.0, 2998.0) |
| **Organism category** |  |  |  |  |  |
| Staphylococcus | 5 (20.0%) | 3 (17.6%) | 4 (20.0%) | 8 (23.5%) | 20 (20.8%) |
| Gram Negative | 2 (8.0%) | 3 (17.6%) | 5 (25.0%) | 7 (20.6%) | 17 (17.7%) |
| Other Gram Positive | 3 (12.0%) | 1 (5.9%) | 1 (5.0%) | 2 (5.9%) | 7 (7.3%) |
| Negative Culture | 3 (12.0%) | 0 (0.0%) | 3 (15.0%) | 2 (5.9%) | 8 (8.3%) |
| No Culture Taken | 0 (0.0%) | 0 (0.0%) | 2 (10.0%) | 0 (0.0%) | 2 (2.1%) |
| Polymicrobial | 12 (48.0%) | 10 (58.8%) | 5 (25.0%) | 15 (44.1%) | 42 (43.8%) |
| **Perioperative antibiotics** |  |  |  |  |  |
| Cefazolin | 19 (76.0%) | 15 (88.2%) | 17 (85.0%) | 31 (91.2%) | 82 (85.4%) |
| Ceftriaxone | 0 (0.0%) | 0 (0.0%) | 3 (15.0%) | 1 (2.9%) | 4 (4.2%) |
| Cefepime | 1 (4.0%) | 0 (0.0%) | 1 (5.0%) | 0 (0.0%) | 2 (2.1%) |
| Ciprofloxacin | 2 (8.0%) | 0 (0.0%) | 0 (0.0%) | 0 (0.0%) | 2 (2.1%) |
| Clindamycin | 3 (12.0%) | 1 (5.9%) | 1 (5.0%) | 2 (5.9%) | 7 (7.3%) |
| Gentamicin | 2 (8.0%) | 1 (5.9%) | 0 (0.0%) | 1 (2.9%) | 4 (4.2%) |
| Vancomycin | 0 (0.0%) | 0 (0.0%) | 4 (20.0%) | 1 (2.9%) | 5 (5.2%) |
| Piperacillin-Tazobactam | 1 (4.0%) | 0 (0.0%) | 0 (0.0%) | 1 (2.9%) | 2 (2.1%) |
| **Return to OR after last planned infection surgery** | 10 (40.0%) | 5 (29.4%) | 9 (45.0%) | 23 (67.6%) | 47 (49.0%) |
