# Supplementary figures and images for: Multisite Study of the Management of Musculoskeletal Infection After Trauma: The MMUSKIT Study
Source: Open Forum Infect Dis. 2024 May 6;11(6):ofae262. doi: 10.1093/ofid/ofae262 (PMC11161894; doi:10.1093/ofid/ofae262)

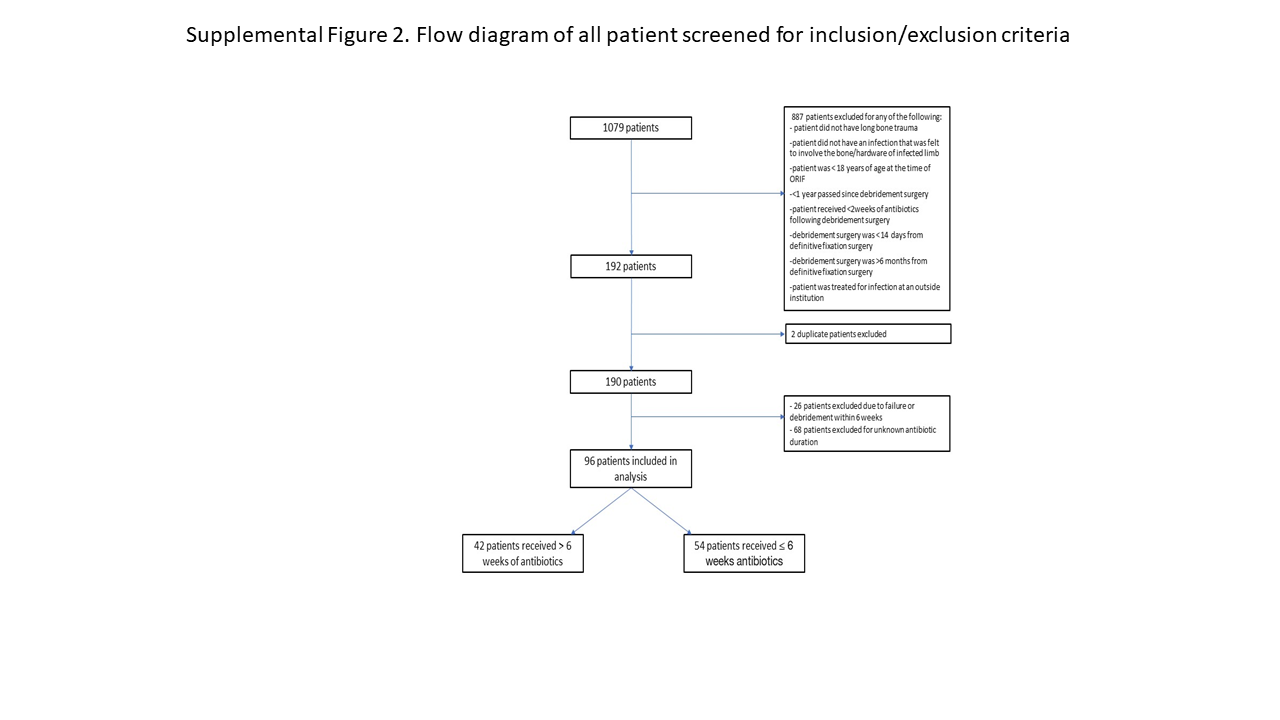

Supplement: ofae262_Supplementary_Data [file ofae262_supplementary_data.zip › supp_Fig_2_300dpi.TIF]
